# Assessment of the physicochemical properties and bacterial composition of *Lactobacillus plantarum* and *Enterococcus faecium-*fermented *Astragalus membranaceus* using single molecule, real-time sequencing technology

**DOI:** 10.1038/s41598-018-30288-x

**Published:** 2018-08-08

**Authors:** Hongxing Qiao, Xiaojing Zhang, Hongtao Shi, Yuzhen Song, Chuanzhou Bian, Aizhen Guo

**Affiliations:** 1 The State Key Laboratory of Agricultural Microbiology, College of Veterinary Medicine, Huazhong Agricultural University, Wuhan, 430070 China; 2 College of Veterinary Medicine, Henan University of Animal Husbandry and Economy, Zhengzhou, 450046 China

## Abstract

We investigated if fermentation with probiotic cultures could improve the production of health-promoting biological compounds in *Astragalus membranaceus*. We tested the probiotics *Enterococcus faecium*, *Lactobacillus plantarum* and *Enterococcus faecium* + *Lactobacillus plantarum* and applied PacBio single molecule, real-time sequencing technology (SMRT) to evaluate the quality of *Astragalus* fermentation. We found that the production rates of acetic acid, methylacetic acid, aethyl acetic acid and lactic acid using *E*. *faecium* + *L*. *plantarum* were 1866.24 mg/kg on day 15, 203.80 mg/kg on day 30, 996.04 mg/kg on day 15, and 3081.99 mg/kg on day 20, respectively. Other production rates were: polysaccharides, 9.43%, 8.51%, and 7.59% on day 10; saponins, 19.6912 mg/g, 21.6630 mg/g and 20.2084 mg/g on day 15; and flavonoids, 1.9032 mg/g, 2.0835 mg/g, and 1.7086 mg/g on day 20 using *E*. *faecium*, *L*. *plantarum* and *E*. *faecium* + *L*. *plantarum*, respectively. SMRT was used to analyze microbial composition, and we found that *E*. *faecium* and *L*. *plantarum* were the most prevalent species after fermentation for 3 days. *E*. *faecium* + *L*. *plantarum* gave more positive effects than single strains in the *Astragalus* solid state fermentation process. Our data demonstrated that the SMRT sequencing platform is applicable to quality assessment of *Astragalus* fermentation.

## Introduction

*Astragalus* root, a well-known traditional herbal medicine, has been widely used in humans, poultry, and other livestock in China and East Asia. It contains polysaccharides, saponins, flavonoids, anthraquinones, alkaloids, amino acids, β-sitosterol, and metallic elements^1–3^. *Astragalus* has anti-inflammatory^4^, immunostimulant^5^, antioxidative^6^ and antiviral activities^7^. Fermentation is often used for various foods, including fruits. However, accumulating evidence has shown that some herbs can also be fermented, for example *Flos Lonicera* and *Rhizoma atractylodis*^8,9^.

Solid state fermentation (SSF) is the process by which microorganisms are cultured on a solid moist substrate. It is considered superior to submerged fermentation technology^10^. SSF has several advantages, including microbial culture on water-insoluble substrates, higher product concentration and stability, higher fermentation productivity, lower catabolic repression, and less need for sterility^10^.

In the literature, quality assessment of fermented *Astragalus* is normally based on changes in microbial composition and physiological parameters, such as pH and water content^11^. However, little is known about organic acid content, yield of active substances, and microbial composition after lactic acid bacterial (LAB) fermentation. Although culture-dependent methods and quantitative real-time polymerase chain reaction (PCR) methods have been used to study microbial composition, these methods are time-consuming and the results are sometimes inaccurate, especially the target bacterial counts^12^.

Recently, there has been a revolution in next-generation sequencing platforms, such as the Sanger sequencing method^13^, which is a high-throughput platform based on the Roche GS20 454 sequencer^14^, Illumina GA, and MiSeq and HiSeq platforms^15^. Since the DNA sequencing techniques can determine only partial sequences of 16 S rRNA genes, these methods have limited taxonomic resolution^16^. Third NextGen, Pacific Biosciences’ (PacBio) single molecule, real-time sequencing (SMRT) technology is now available, which is faster and provides more informative sequencing. PacBio offers long DNA sequence reads that are able to depict the bacterial profiles of target samples to the species level^17^.

The PacBio SMRT method has been used to evaluate the quality of silage production successfully^16^. In the present study, we specifically focused on the detection and comparison of the bacterial composition of *Astragalus* fermented by *Enterococcus faecium* and *Lactobacillus plantarum* using the PacBio SMRT method.

## Results

### pH changes during *Astragalus* fermentation

**Figure 1:**
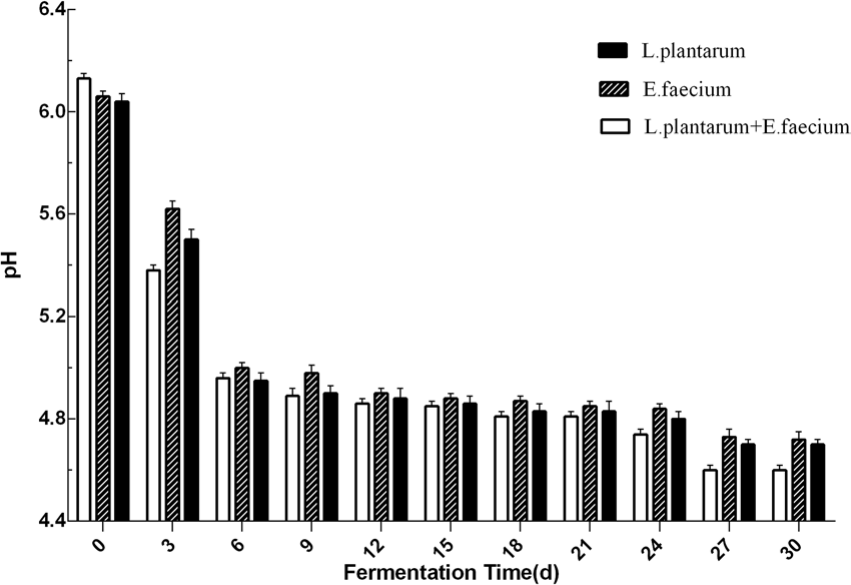
Determination of pH using *E*. *faecium*, *L*. *plantarum*, or *L*. *plantarum* + *E*. *faecium*. Samples were taken for analysis after 0, 3, 6, 9, 12, 15, 18, 21 24, 27, and 30 days. Data are expressed as the mean ± SD from three independent experiments.

The changes in pH of fermented *Astragalus* are shown in Fig. 1. Generally, the addition of one or two LAB additives (*L*. *plantarum*, *E*. *faecium*, *L*. *plantarum* + *E*. *faecium*) resulted in varying degrees of fermentative pH changes. After fermentation, pH decreases to below 5.0 were significant from days 6 to 30 in the *L*. *plantarum*, *E*. *faecium and L*. *plantarum* + *E*. *faecium groups*.

### Changes in organic acid content during fermentation

#### Acetic acid analysis

**Figure 2:**
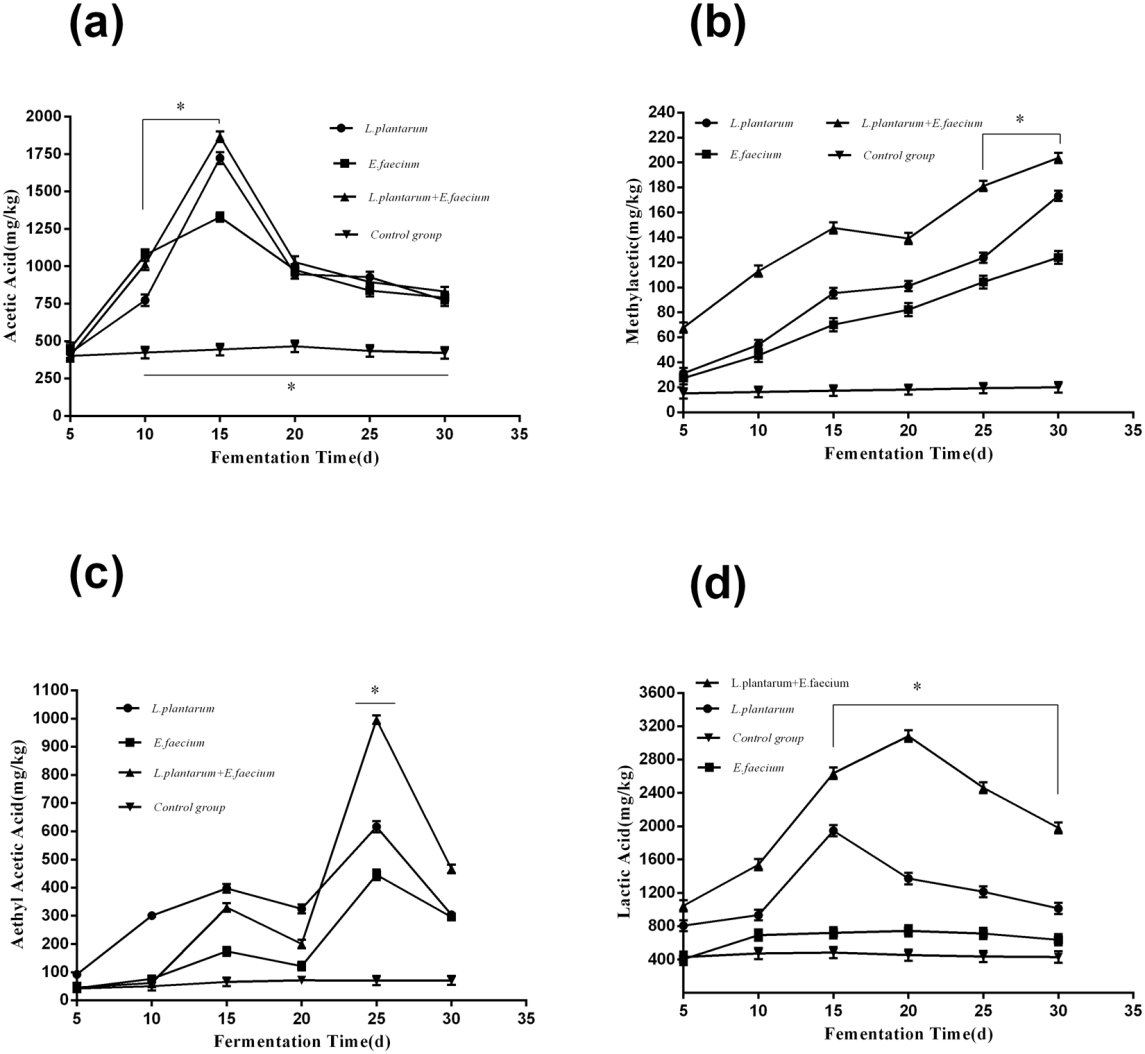
Determination of organic acids in *Astragalus* fermented using *L*. *plantarum*, *E*. *faecium*, or *L*. *plantarum* + *E*. *faecium* compared to the control group. Samples were taken for analysis after 5, 10, 15, 20, 25, and 30 days. Data are expressed as the mean ± SD from three independent experiments. (**a**): Acetic acid analysis; (**b**): Methylacetic analysis; (**c**): Aethyl acetic acid analysis; and (**d**): Lactic acid analysis. Boxes with one asterisk indicate significant differences at P < 0.05 compared with control group.

There were significant rises in acetic acid content in the *L*. *plantarum group*, *E*. *faecium group*, *and L*. *plantarum* + *E*. *faecium* group, reaching peaks of 1723.01 mg/kg, 1329.61 mg/kg, and 1866.24 mg/kg on day 15, respectively, as shown in Fig. 2(a). However, there was no significant change in the control group. These results demonstrated that *L*. *plantarum and E*. *faecium* promoted the production of acetic acid.

#### Methylacetic acid analysis

There was a gradual rise in methylacetic acid in the *L*. *plantarum* group, *E*. *faecium* group, and *L*. *plantarum* + *E*. *faecium* group, reaching peaks of 173.29 mg/kg, 123.88 mg/kg, and 203.80 mg/kg on day 30, respectively, as shown in Fig. 2(b). The production of methylacetic acid may continue to increase with the extension of fermentation. These results suggest that *L*. *plantarum* and *E*. *faecium* promoted the production of methylacetic acid.

#### Aethyl acetic acid analysis

There were significant rises in aethyl acetic acid in the *L*. *plantarum* group, *E*. *faecium* group, and *L*. *plantarum* + *E*. *faecium* group, reaching peaks of 616.07 mg/kg, 445.74 mg/kg, and 996.04 mg/kg on day 25, respectively, as shown in Fig. 2(c). These results indicated that *L*. *plantarum* and *E*. *faecium* promoted the production of aethyl acetic acid.

#### Lactic acid analysis

There were gradual rises in lactic acid in the *L*. *plantarum* + *E*. *faecium* group and *L*. *plantarum* group, reaching peaks of 3081.99 mg/kg on day 20 and 1946.17 mg/kg on day 15, respectively, as shown in Fig. 2(d). However, the *E*. *faecium* group exhibited no significant change compared to the control group. These results indicated that *L*. *plantarum* + *E*. *faecium* promoted the production of lactic acid.

### Active substance yields in fermented Astragalus

#### Polysaccharide yield analysis

**Figure 3:**
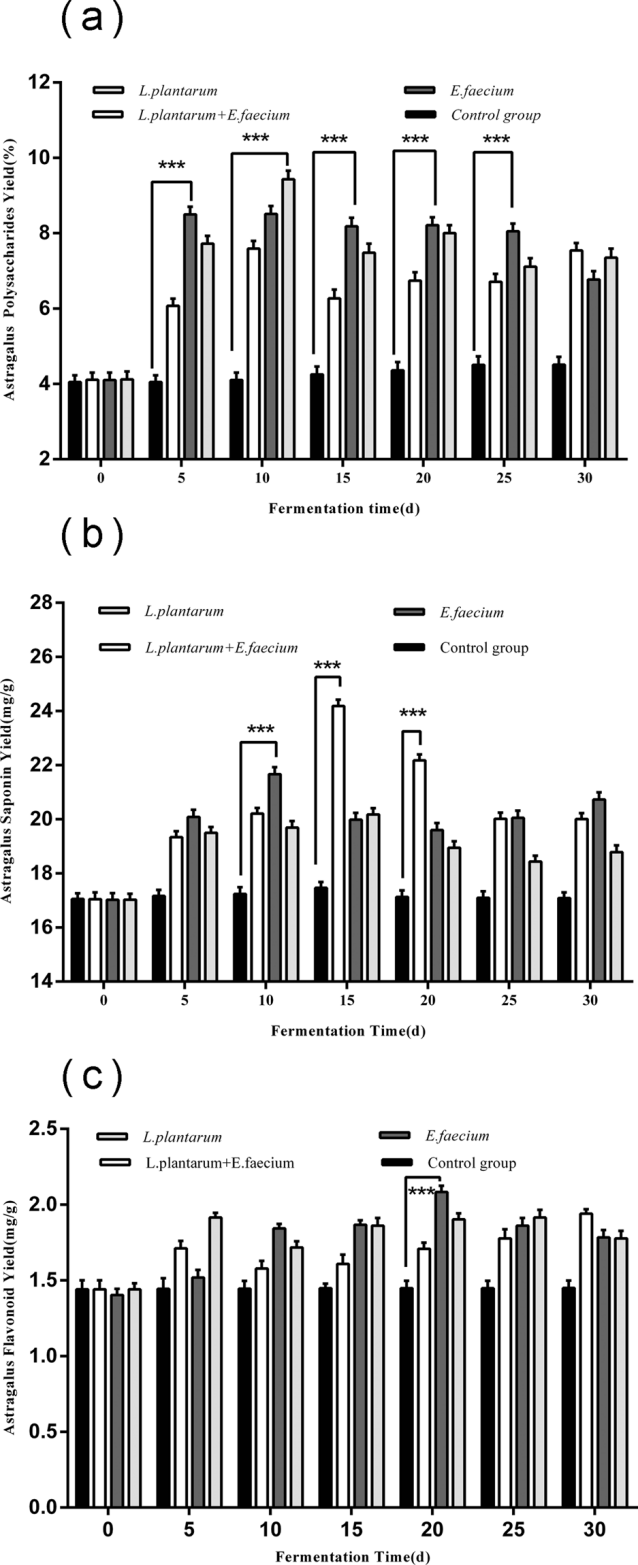
Analysis of active substance yields of *Astragalus* fermented using *L*. *plantarum*, *E*. *faecium*, or *L*. *plantarum* + *E*. *faecium* compared to the control group. Samples were taken for analysis after 0, 5, 10, 15, 20, 25, and 30 days. Data are expressed as the mean ± SD from three independent experiments. Boxes with three asterisk indicate very significant differences at P < 0.01 compared with control group.

The polysaccharide yield was higher in the *L*. *plantarum* fermentation than in the control group as shown in Fig. 3(a). Moreover, the polysaccharide yield was 9.43% on day 10, which was 2.3-fold higher than that of the control group, and it remained steady reaching a maximum on day 30. These results indicated that the polysaccharide yield changed significantly by *L*. *plantarum* fermentation (p < 0.05).

#### Total saponins yield analysis

The total yield of saponins in the *L*. *plantarum* + *E*. *faecium* group sharply increased on day 15 and reached 21.6630 mg/g as shown in Fig. 3(b), representing a 125.68% increase compared to the control group. These results meant that the combination of *L*. *plantarum* + *E*. *faecium* was superior to either *L*. *plantarum* or *E*. *faecium* alone (p < 0.05).

#### Flavonoid yield analysis

The flavonoid yield in the *E*. *faecium* group sharply increased and reached 2.0835 mg/g as shown in Fig. 3(c). Moreover, this was 1.44-fold higher than the yield for the control group. There seemed to be a tendency for the flavonoid yield to reach two peaks, during the initial and later stages of the fermentation. These results revealed that the flavonoid yield changed during *E*. *faecium* fermentation (p < 0.05).

#### Changes in microbial composition after Astragalus fermentation

SMRT sequencing of the full-length 16 S rRNA genes was performed to obtain accurate bacterial profiles of the *Astragalus* samples at the species level. A total of 2,945,166 sequence reads were obtained from nine *Astragalus* samples, with an average of 8,888 reads for each sample. The ACE, Chao 1, Shannon and Simpson indices were calculated, and a different richness for each of the nine groups was observed (Table 1). These results indicated that the samples showed high bacterial biodiversity.

**Table 1:** Diversity estimation of the 16 S rRNA gene libraries of the nine samples from the sequencing.

Sample	ACE	Chao 1	Shannon	Simpson
A1	60.07	29	1.18	0.51
A2	53.19	31	1.44	0.55
A3	249.41	178	4.9	0.92
B1	87.7	27	1.19	0.52
B2	224.59	79	2.35	0.72
B3	321.63	190	4.95	0.92
C1	54.53	31	1.21	0.52
C2	167.45	110	2.51	0.67
C3	294.69	178	4.89	0.93

Note: A1: *Astragalus* samples fermented using *E*. *faecium* on day 0; A2: *Astragalus* samples fermented using *E*. *faecium* on day 3; A3: *Astragalus* samples fermented using *E*. *faecium* on day 30; B1: *Astragalus* samples fermented using *L*. *plantarum* on day 0; A2: *Astragalus* samples fermented using *L*. *plantarum* on day 3; A3: *Astragalus* samples fermented using *L*. *plantarum* on day 30; C1: *Astragalus* samples fermented using *E*. *faecium* + *L*. *plantarum* on day 0; C2: *Astragalus* samples fermented using *E*. *faecium* + *L*. *plantarum* on day 3; C3: *Astragalus* samples fermented using *E*. *faecium* + *L*. *plantarum* on day 30.

**Figure 4:**
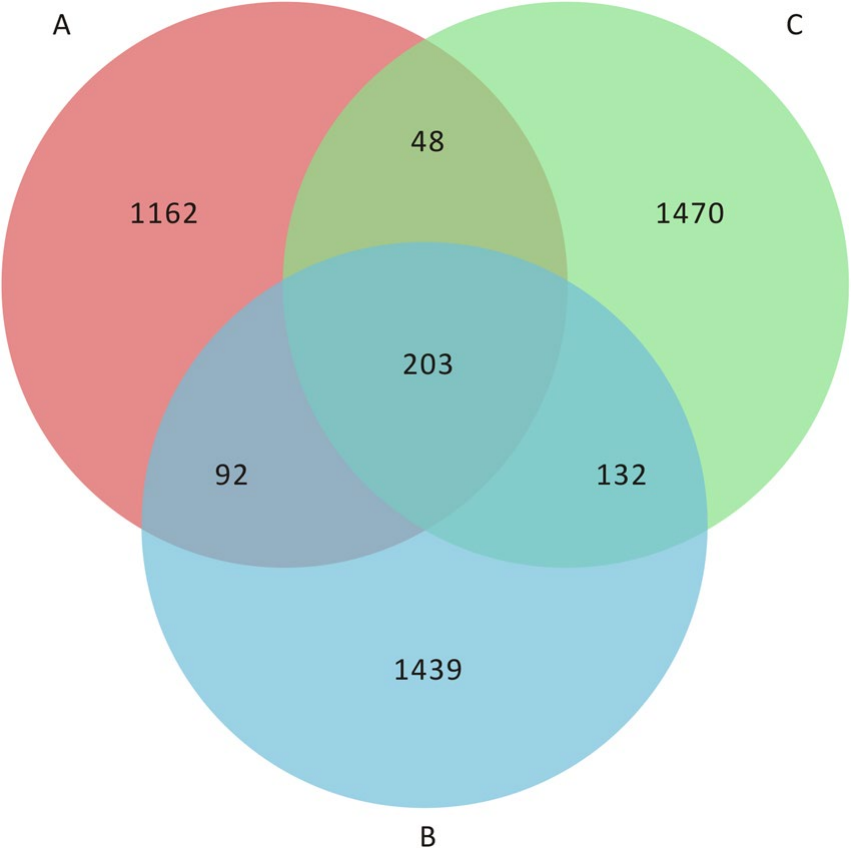
Shared OTU analysis of the different groups. The number of species in the A group is 1505; the number of species in the B group is 1866; the number of species in the C group is 1853; the number of species common to the A and B groups is 295; the number of species common to the A and C groups is 251; the number of species common to the B and C groups is 335; a total of 203 OTUs were common to the three groups.

The total OTUs obtained were as follows: 1,505 in the A group (*E*. *faecium* fermentation), 1,866 in the B group (*L*. *plantarum* fermentation), and 1,853 in the C group (*E*. *faecium* + *L*. *plantarum* fermentation). As shown in Fig. 4, a total of 203 OTUs were common among the three groups, whereas the number of OTUs present only in one group varied from 1,162 to 1,470.

#### Microbial comparative analysis

**Figure 5:**
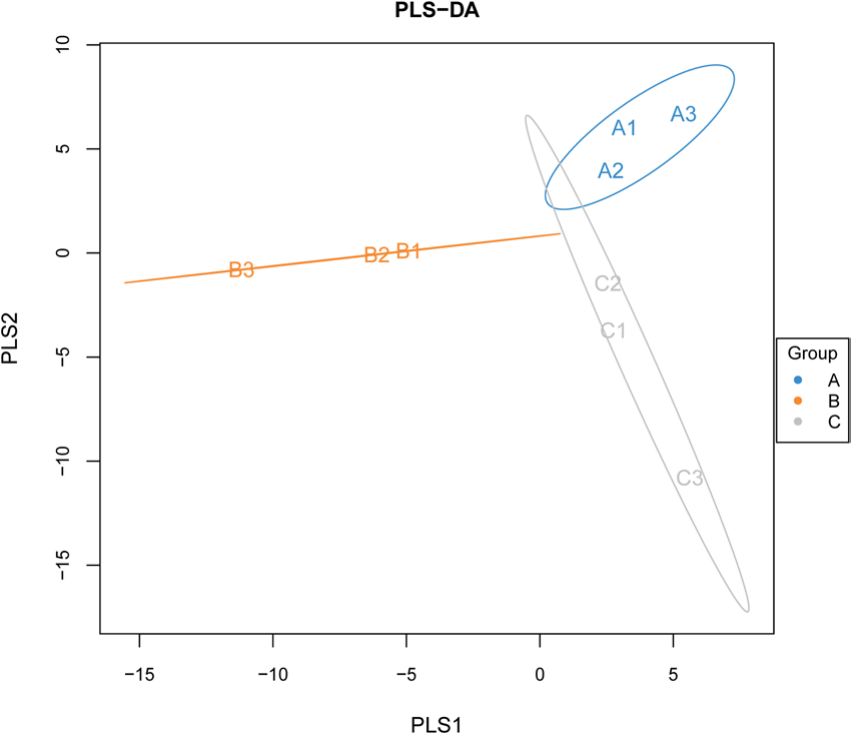
PLS-DA discriminant model from A1, A2, A3, B1, B2, B3, C1, C2, and C3 samples. VIP coefficients of A1, A2, A3, C1, C2, and C3 were greater than 1; however, those of B1, B2, and B3 were less than 1.

As shown in Fig. 5, the variable importance in projection (VIP) coefficient was calculated for each species. The results showed that the VIP coefficients of A1, A2, A3, C1, C2, and C3 were greater than 1; however, the VIP coefficients for B1, B2, and B3 were less than 1. These results showed that the shorter the distance between the same groups, the further the distance between the different groups, indicating that the classification model works well.

#### Bacterial community composition

**Figure 6:**
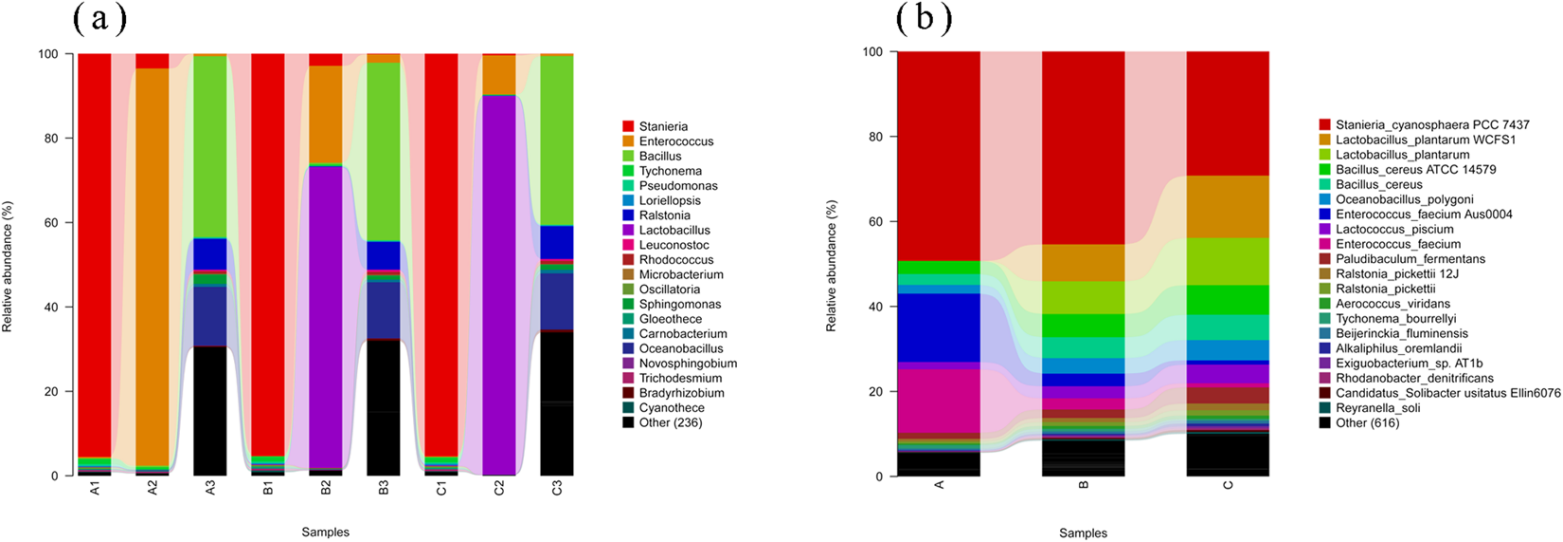
Genus-level and species-level analysis of the samples. (**a**) Genus-level analysis of the nine samples. Overall microbiota composition of fermentation samples at the genus level for: A1, on day 0 using *E*. *faecium*; A2, on day 3 using *E*. *faecium*; A3, on day 30 using *E*. *faecium*; B1, on day 0 using *L*. *plantarum*; B2, on day 3 using *L*. *plantarum*; B3, on day 30 using *L*. *plantarum*; C1, on day 0 using *L*. *plantarum* + *E*. *faecium*; C2, on day 3 using *L*. *plantarum* + *E*. *faecium*; and C3, on day 30 using *L*. *plantarum* + *E*. *faecium*. The relative abundances of *E*. *faecium* and *L*. *plantarum* are shown on the *y*-axis. (**b**) Species-level analysis of the three groups. A: Overall microbiota composition of fermentation samples at the species level using *E*. *faecium*. B: Overall microbiota composition of fermentation samples at the species level using *L*. *plantarum*. C: Overall microbiota composition of fermentation samples at the species level using *L*. *plantarum* + *E*. *faecium*. The relative abundances of *E*. *faecium* and *L*. *plantarum* are shown on the *y*-axis.

As shown in Fig. 6(a), an analysis of the most abundant taxa at the genus level revealed that *E*. *faecium* (94.0%) was most abundant in the A2 sample, *L*. *plantarum* (71.0%) in the B2 sample, and *E*. *faecium* + *L*. *plantarum* (98.7%) in the C2 sample. However, microbial compositions in the A3, B3, and C3 samples tended to be more consistent at day 30. These results showed that the genus *Stanieria* spp. was the most abundant in the A1, B1, and C1 samples before fermentation, whereas after fermentation on day 3, *Enterococcus* and *Lactobacillus* were the most abundant genera in the A2, B2, and C2 samples.

At the species level, the relative abundances of microbes in the three groups are shown in Fig. 6(b). *E*. *faecium* exhibited dynamic changes in the A group, and its proportion was 44.8%. *L*. *plantarum* displayed slight changes in the B group, and its proportion was 35.3%. *E*. *faecium* + *L*. *plantarum* underwent great changes in the C group, and its proportion was 47.35%. Clearly, the prevalent species that existed in fermented *Astragalus* were highly dependent on the original bacterial composition.

#### Community compositional heat map combined with cluster analysis

**Figure 7:**
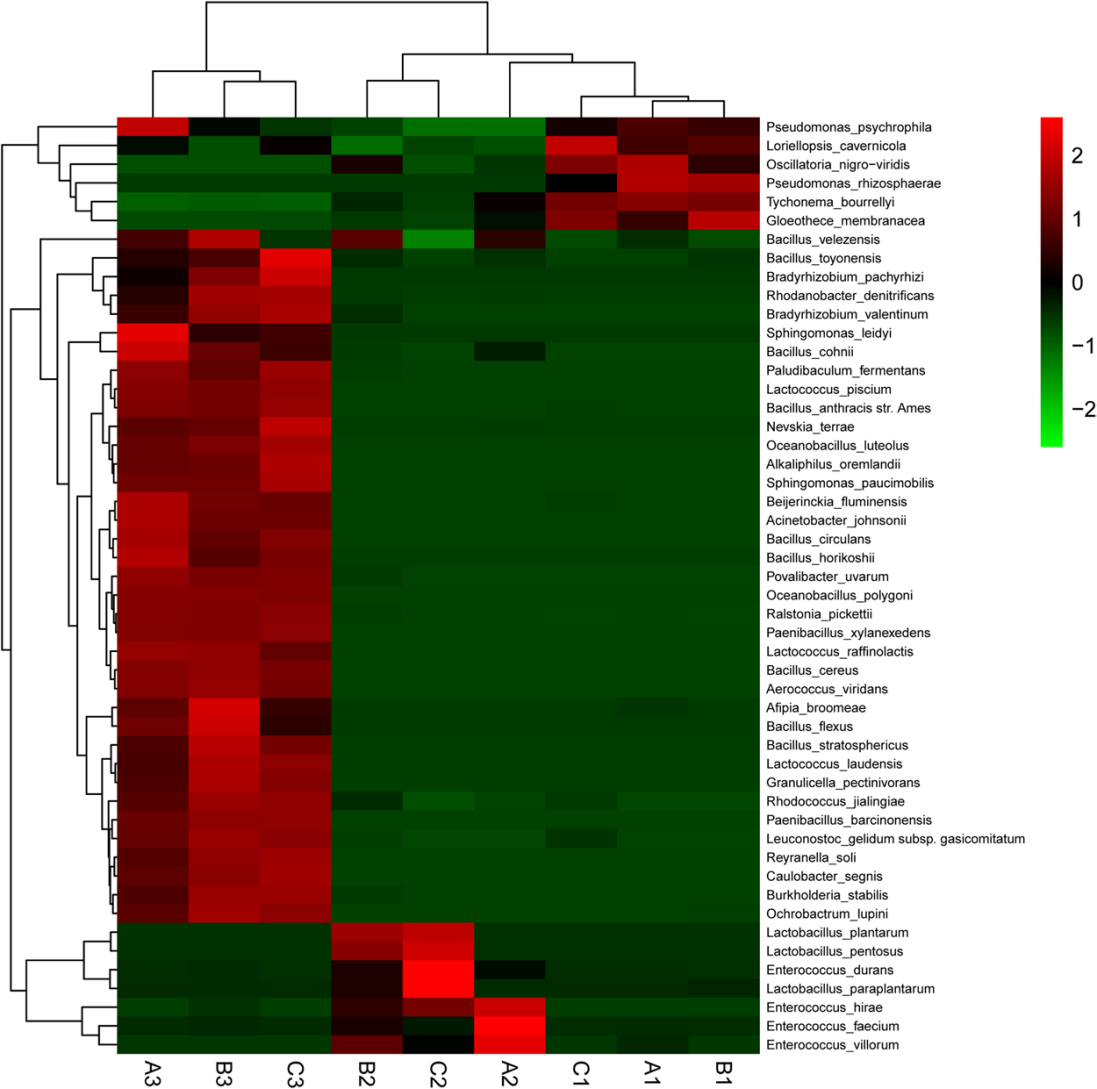
Heat map analysis of the nine samples. Heat map showing that the abundances of the top 50 species are clustered was plotted using R software. Red represents species with higher abundances in the corresponding sample, and green represents species with lower abundances. *Enterococcus* and *Lactobacillus* were present at higher abundances in groups A2, B2, and C2 than in the other groups.

As shown in Fig. 7, the top 50 species according to abundance were clustered and plotted using R software. Red represents species with higher abundance in the corresponding samples, and green represents species with lower abundance. From the heat map, it can be seen that *Enterococcus* and *Lactobacillus* were more abundant in the A2, B2, and C2 samples than in the other samples. However, with time the fermentation bacteria were reduced in numbers and other naturally occurring bacteria commenced growth.

On day 30 of the fermentation, we concluded that the fermentation bacteria were dominant on day 3 of the fermentation, and this indicated that 3 days may be the optimum length of time for *Astragalus* fermentation.

## Discussion

The Chinese herb *A*. *membranaceus* (*Astragalus*) has been widely used as a dietary supplement in Asia^18^. In this study, *E*. *faecium* and *L*. *plantarum* were added to assess the nutritional value and organic content of *Astragalus* fermented with probiotics. The full 16 S rRNA gene-SMRT sequencing method was applied to monitor the quality of *Astragalus* fermentation, as traditional methods, including culture-dependent methods, can be inaccurate and the results may be ambiguous and difficult to interpret.

In this study, we found that the fermentation of *Astragalus* with *E*. *faecium* and *L*. *plantarum* additives caused a decrease in pH due to the production of organic acids during the fermentation process. In general, the decrease in pH value was mainly due to the production of lactic acid during fermentation. The low pH is advantageous because it creates a more stable fermentation. The organic acid production of fermented *Astragalus* is dependent upon the type of bacteria used. *L*. *plantarum* is a well-known homo-fermentative LAB^19^, which efficiently produces lactic acid from fermented *Astragalus*. Moreover, the fermentation conducted with combined *E*. *faecium* + *L*. *plantarum* was enhanced compared to single-strain fermentation. It has been reported that there is a natural synergy between different probiotics when they are present in certain proportions^20,21^. It is important to determine the appropriate ratios of bacteria because unsuitable proportions can reduce the rates of production of organic acids and perhaps even inhibit the fermentation process^22^. Also, combining *E*. *faecium* + *L*. *plantarum* in the fermentation may improve the odor and provide an acidic flavor^16^.

Moreover, the current study demonstrated that *Astragalus* produces polysaccharides, flavonoids and saponins, which increased gradually during fermentation and were found in higher concentrations than in the control group during the entire fermentation process when *E*. *faecium* and *L*. *plantarum* were applied to SSF. The reason may be that free polysaccharides and extracellular polysaccharides were produced due to the degradation of cellular wall cellulose by digestive enzymes during fermentation^23^. At the later stage of fermentation, the yield of active substances decreased, which may be due to their utilization by *E*. *faecium* and *L*. *plantarum*, their transformation into other compounds, or the generation of secondary glycosides.

The microbial profile is another indicator that reflects the quality of fermented *Astragalus*. The current study focused on the microbial composition during fermentation of *Astragalus* powder using *E*. *faecium* and *L*. *plantarum*. The results showed that *Astragalus* may promote the growth of *E*. *faecium* and *L*. *plantarum* because of the high content of organic acids, carbon, inorganic salts, and polysaccharides^24,25^. The preparation of traditional Chinese medicines mostly incorporates extraction and prefractionation processes^26^ that can be accompanied by an irritating odor^27^ which is not conducive to animal feeds. Thus, the fermentation of herbal medicines by probiotics may be an effective processing method to remove odor^28^. Moreover, *Astragalus* has a sour taste after fermentation which is more conducive to animal feeds.

SMRT analysis of the microbial composition of nine fermented *Astragalus* samples showed that the major bacterial species depended on the original bacterial composition. After day 3 of fermentation, the original bacteria namely *E*. *faecium* and *L*. *plantarum*, were dominant. Moreover, the microbial diversity was greater when *E*. *faecium* + *L*. *plantarum* were used for fermentation compared to a single species. These results confirmed that multiple strains have positive effects on *Astragalus* fermentation. In conclusion, it appears that *Astragalus* promoted the growth of bacteria to exert prebiotic-like effects that selectively stimulated the growth of synergistic beneficial bacteria^29^.

However, microbial composition tended to remain constant after fermentation for 30 days. The reason is that lactic acid bacteria are relatively fragile, with a short growth period^30,31^. By 30 days, bacteria naturally associated with *Astragalus* had begun to grow and multiply. Compared with the traditional method of colony counting^32^, SMRT analysis can accurately reflect microbial composition during *Astragalus* fermentation.

In summary, *E*. *faecium* and *L*. *plantarum* have positive effects on the fermentation of *Astragalus*. Although only nine of the samples were analyzed using the SMRT sequencing technology, our data show that this is a promising method for assessing the quality of fermented *Astragalus*.

## Materials and Methods

### Preparation of fermented *Astragalus*

*Astragalus*, the dried root of *Astragalus membranaceus* (Fisch.) Bge. var. mongholicus was obtained from Gansu Hui Sen Pharmaceutical Co., Ltd. (Minxian, Gansu, China). *Astragalus* fermentation was performed following our laboratory-optimized procedure. Briefly, *Astragalus* was ground into a powder using a 100-mesh screen. Then, the dried powder (7,500 g) was divided into three groups: A, B, and C. The A group was inoculated with 10^6^ colony-forming units (CFU)/g of *E*. *faecium* (CGMCC 1.130), the B group was inoculated with 10^6^ colony-forming units (CFU)/g of *L*. *plantarum* (CGMCC 1.557), and the C group was inoculated with 10^6^ CFU/g *E*. *faecium* + *L*. *plantarum*. The two strains were isolated and deposited in the China General Microbiological Culture Collection Center (CGMCC, Beijing, China). The control group did not add any probiotics. Fermentation was conducted in 35 × 45-mm plastic film bags (Jinhu Co., Zhejiang, China), and the bags were evacuated and sealed using a vacuum packing machine. Subsequently, the mixtures were incubated for 30 days at 37 °C under anaerobic conditions to produce *Astragalus* fermentation. The three groups were sampled at days 0, 3, and 30 and labeled A1, A2, A3, B1, B2, B2, C1, C2, and C3, respectively.

### Changes in organic acid contents and pH value during fermentation

To perform organic acid analysis, 5 g of day 5, 10, 15, 20, 25 and 30 fermented sampls from A group, B group, C group and control group were mixed with 60 mL of deionized water, followed by heating in a water bath for 20 min. Then, the filtrate was centrifuged at 80,000 × *g* for 20 min. Ten milliliters of supernatant were filtered through a 0.45-μm membrane before chromatographic analysis. Separations by high performance liquid chromatography (HPLC) were performed on an Agilent 1260 Series LC system with a preparative XB-C18 column (4. 6 mm × 150 mm, i.d. 5 μm, Waters, USA). Solvent A was phosphate buffer solution (pH 2.70), and solvent B was methanol solution. Elution was performed with a gradient of 97:3, while the analytical column temperature was 20 °C, and the flow rate was 0.80 mL/min. Absorbance was detected at 210 nm. Acetic acid, methylacetic acid, aethyl acetic acid, and lactic acid concentrations were determined. For pH measurement, 25 g fermented *Astragalus* from the three groups was dissolved in 225 mL of deionized water. After vortex mixing for 30 min, measurements were taken using a pH meter (Mettler Toledo, Greifensee, Switzerland).

### Active substance analysis of fermented *Astragalus*

#### Astragalus polysaccharide yield analysis

Fermented dried *Astragalus* root was kept in distilled water (at a ratio of 1:8) for 24 h and extracted 3 times with distilled water in a boiling water bath. The extract was collected by centrifugation (Xiangyi, Changsha, China) at 5, 000 × *g* for 15 min, and the supernatant was concentrated by rotary evaporation (Yarong, Shanghai, China). Then, 95% ethanol (3-fold volume) was added to the concentrated solution and the mixture was stored at 4 °C for 24 h and then centrifuged at 5, 000 × *g* for 20 min. The precipitate was dried at 60 °C and ground into a powder. The amounts of polysaccharides in the extracts were determined using the phenol-sulfuric acid method^3^.

#### Total yield analysis of saponins

*Astragalus* methyl glucoside reference substance (Suolaibao Co., Shanghai, China) (5 mg) was added to a 25-mL volumetric flask, and methanol was added, diluted to scale, and the mixture was shaken well and used as a reference solution. There were 0.1-, 0.2-, 0.4-, 0.6-, 0.8-, 1.0-, and 1.2-mL aliquots of the reference solution that were placed in 10-mL calibrated test tubes, dried in a water bath, and cooled. Then, 0.2 mL of freshly prepared 5% vanillin glacial acetic acid solution was added to 0.8 mL of perchloric acid and shaken well. After that, the mixture was heated at 70 °C in a water bath for 20 min, cooled with ice water for 5 min, and followed by the addition of 5 mL of glacial acetic acid. The mixture was shaken well and retained as a blank control. A standard curve was constructed with absorbance at 580 nm as the ordinate (Y) and the concentration of reference solution as the ordinate (X). The production of glycosides from *Astragalus* could be calculated according to the standard curve.

#### Flavonoid yield analysis

A 10 mg sample of a rutin reference solution (Suolaibao Co.) was mixed with 60% ethanol to a final volume of 50 mL in a volumetric flask and used as the reference solution. 0-, 2-, 4-, 6-, 8-, 10-, and 12-mL aliquots of the reference solution were placed in 25-mL volumetric flasks, and then 1.0 mL of 5% sodium nitrite solution was added and shaken well for 6 min. After that, 1.0 mL of 10% aluminum nitrate solution was added and shaken well for 6 min, then 10 mL of 4% sodium hydroxide solution in 60% ethanol was added and shaken for 15 min. A standard curve was constructed with absorbance at 510 nm as the ordinate (Y) and concentration of the reference substance as the ordinate (X), and the production of astragaloside IV was calculated according to the standard curve.

Flavonoids yield = [flavonoid concentration (mg/mL) × flavonoid solution volume (mL)]/sample quality (g) × 100%.

#### SMRT analysis of microbial composition

A total of nine samples, A1, A2, A3, B1, B2, B3, C1, C2, and C3, were collected. The samples were immediately frozen at −196 °C until DNA extraction. A total of 200 mg of fermented *Astragalus* from each group was utilized for DNA isolation. DNA samples were quantified using a Qubit 2.0 Fluorometer (Invitrogen, Carlsbad, CA, USA). The quality of extracted DNA was assessed by 0.8% agarose gel electrophoresis and spectrophotometry (optical density at 260 nm/280 nm). All of the extracted DNA samples were stored at −20 °C prior to further analysis.

Bacterial 16 S rRNA was amplified by PCR for barcoded SMRT sequencing with forward 27 F (5′-AGAGTTTGATCMTGGCTCAG-3′) and reverse 1492 R (5′-ACCTTGTTACGACTT-3′) primers. The PCR program was as follows: 95 °C for 2 min; 30 cycles of 95 °C for 30 s, 55 °C for 30 s and 72 °C for 30 s with a final extension of 72 °C for 5 min.

The entire 16 S rRNA lengths of the community were sequenced by the Pacbio Sequel platform at Personalbio, Inc. (Shanghai, China). The raw data were taken for Circular Consensus Sequencing and corrected so that the correctness of the forecast was not less than 90%. The extraction of high-quality sequences was performed with the QIIME package (Quantitative Insights into Microbial Ecology, v1.8, http://qiime.org/acripts/pick_oyus.html), and they were clustered into operational taxonomic units (OTUs). All of the sequences were compared against the Greengenes reference database (release 13.8, http://greengenes.secondgenome.com/). Taxa summarization, alpha diversity, and a taxon differential distribution analysis were performed using all of the available sequences for each sample.

Alpha diversity was estimated using the Chao 1, ACE, Shannon and Simpson indices. The Chao1 richness estimation index estimates the number of species actually present in the community by calculating the number of OTUs that were detected only once or twice in the community. The ACE richness estimation index takes into account the OTUs with a sequence size of 10 to estimate the number of species actually present in the community. The Shannon diversity index comprehensively considers the richness and evenness of the community. The Simpson diversity index is a common index for evaluating community diversity. The larger the Chao1 and ACE indices, the higher the richness of the community, and higher Shannon and Simpson index values, indicate that higher community diversity. Simultaneous use of the four indicators clearly demonstrated the bacterial diversity of the 9 samples in this study. The raw sequence reads have been deposited in the National Center for Biotechnology Information Short Read Archive under accession numbers SAMN07411593-SAMN07411601.

#### Statistical analysis

Experimental data were analyzed using Prism software (Graphpad), and statistical significance was tested using analysis of variance. The chemical composition of each sample was tested three times, and the results are expressed as the mean ± standard deviation.

Sequences were rarefied prior to the calculation of alpha and microbial comparative analysis. Alpha diversity indices were calculated in QIIME from rarefied samples using the Shannon index for diversity and the Chao 1 index for richness^16^. Using R software, a partial least squares discriminant analysis (PLS-DA) discriminant model was constructed based on the species abundance matrix and the sample packet data.
